# Structural optimization and flow field analysis of turbomolecular pump based on a new performance prediction algorithm

**DOI:** 10.1038/s41598-024-63690-9

**Published:** 2024-06-03

**Authors:** Kun Sun, Haishun Deng, Cheng Wang, Shiwei Zhang

**Affiliations:** 1 School of Mechatronics Engineering, Anhui University of Science and Technology, Huainan, 232001 Anhui China; 2 Anhui Intelligent Mine Technology and Equipment Engineering Research Center, Anhui University of Science and Technology, Huainan, 232001 Anhui China; 3 School of Mechanical Engineering & Automation, Northeastern University, Shenyang, 110819 Liaoning China

**Keywords:** Aerospace engineering, Mechanical engineering

## Abstract

In the present study, a new turbomolecular pump (TMP) performance prediction algorithm is proposed according to the variable surface combined blade row (VSCBR) geometric model. The simulation calculation program is designed to perform structural optimization and flow field analysis. Research on the pumping performance of the traditional straight blade row (TSBR) indicates that when the blade velocity ratio is greater than 1, the increase in the pumping speed and compression ratio of the TMP gradually tends to stabilize. Response surface methodology is used to optimize the structural parameters of the first four stages of the combined blade row. The optimized VSCBR increases the pumping speed by 18.2% compared to that of the TSBR. The flow field analysis based on the optimized VSCBR shows that gas molecules reaching the rear blades are likely to approach the outlet, and the proportion of gas molecules in this region exceeds 50%. Therefore, the blades we designed should be conducive to additional gas molecules reaching the outlet.

## Introduction

Turbomolecular pumps (TMPs) are the main equipment used for obtaining and maintaining high or ultrahigh vacuum environments; they have various advantages, including simple operation, fast start-up, and no oil pollution, and they are widely applied in many applications, such as wafer fabrication, the integrated circuit industry, vacuum coating and surface processing technology^1–3^. The position and role of TMPs in the development of modern high-end industry are very important.

With the rapid development of magnetic levitation bearings and high rotational speed electrical machinery technology^4–6^, these bearings have been successfully applied in TMPs, which has increased the rotational speed of turbine rotors. This increase has increased the rotational speeds of TMPs from thousands of revolutions per minute to tens of thousands of revolutions per minute. The linear velocity of the blades is very close to or even greater than the average thermal motion velocity of the gas molecules. An increase in the turbine rotor speed should significantly improve the pumping performance of the TMP, but the current growth trend is not obvious. From a structural perspective, the blade row structure of the turbine rotor still uses the traditional straight blade row (TSBR) with equal widths and thicknesses of the top teeth^7^, which cannot match its high rotational speed. From a theoretical model perspective, traditional modelling methods are mainly based on past single-stage or two-stage blade row models^8^, which cannot accurately reflect the transport process of gas molecules in multistage blade rows. These two main factors constrain the improvement in the pumping performance of TMPs.

Although the TSBR structure has the advantage of being easy to manufacture and process, it does not match the turbine rotor at high rotational speeds. The linear velocity ($V = rw$) of the blades gradually increases along the radial ($r$) direction, and the most probable velocity ($V_{{{\text{mp}}}} = \sqrt {2RT/M}$) is constant for the same type of gas molecule. Therefore, the high rotational speed increases the difference in the blade velocity ratio ($c = V/V_{{{\text{mp}}}} = r\omega /\sqrt {2RT/M}$) between the turbine rotor at the root ($r = a\_r_{r}$) and at the tip ($r = a\_r_{t}$). The fixed inclination and equal thickness parallel plate structure (namely, the TSBR structure) limits the improvement in the pumping performance.

The traditional geometric structure model was established according to the single-stage or two-stage blade row. First, Schneider^9^ and Heo^10^ used simple geometric structures. These scholars evaluated the pumping speeds and compression ratios of single-stage blade rows based on two-dimensional models. With the popularization and application of direct simulation Monte Carlo methods (DSMCs), Chang^11^ and Li^12^ used three-dimensional models to study pumping characteristics. Later, Amoli^13^ and Hosseinalipour^14^ studied the characteristics of gas molecules in rotor–stator rows. The blade thickness was considered. The simulation results were consistent with the experimental results. However, these scholars did not investigate the multistage combined blade row model, establish detailed region boundary conditions or consider the changes in the blade inclination angle and blade thickness with respect to the radius in the blade equations. Shams^15^ compared the simulation results of nonparallel and parallel blades by employing the test particle Monte Carlo (TPMC) method. However, the blade thickness was not considered, and the simulation calculation algorithm was not described in detail. Moreover, many scholars^16–18^ have focused on magnetic levitation bearings, pumping speed, and compression ratio; structural optimization and flow field analysis of TMPs have rarely been studied at high rotational speeds.

Therefore, it is urgent to propose a new variable surface combined blade row (VSCBR) structure to overcome the limitation of increasing pumping speed. It is very valuable to establish a new modelling method and performance prediction algorithm to accurately evaluate the pumping performance of TMPs and improve their pumping speed.

This work is developed on the basis of previous research^19^, and there are obvious differences between them. In this work, the rotor rows and the stator rows are partitioned into 8 regions instead of 6 regions to study the pumping process of the VSCBR and track gas molecule motion trajectories inside the TMP. In terms of geometric modelling, a three-dimensional geometric model is established according to the real geometric structure of the multistage blade row instead of the two-stage blade rows (a rotor–stator row). The blade thickness and the gap between the rotor rows and stator rows are considered. The above improvement measures closely reflect the actual working conditions of the TMP and increase the accuracy of the simulation calculations.

In addition, the parameters of the blade root angle, blade tip angle, blade root thickness and blade tip thickness are introduced into the blade equations for the first time. This introduction is a prerequisite for performing the structural optimization of the TSBR and the flow field analysis of the VSCBR. The equation of each blade and the gas molecule flight time from one region to another region are solved in the Cartesian coordinate system. A simulated calculation program is designed for the VSCBR based on the new performance prediction algorithm. The experimental testing and verification processes are conducted by using the traditional TMP. The simulation results and experimental results are compared, and the results are consistent. By taking the first four-stage combined blade row that has a significant impact on the pumping speed as an example, the pumping performance of the TSBR with respect to the blade velocity ratio is studied according to our designed program. The structural parameters of the TSBR are optimized by using response surface methodology, and flow field analysis is carried out on the optimized structural parameters.

## Establishing the calculation model

**Figure 1:**
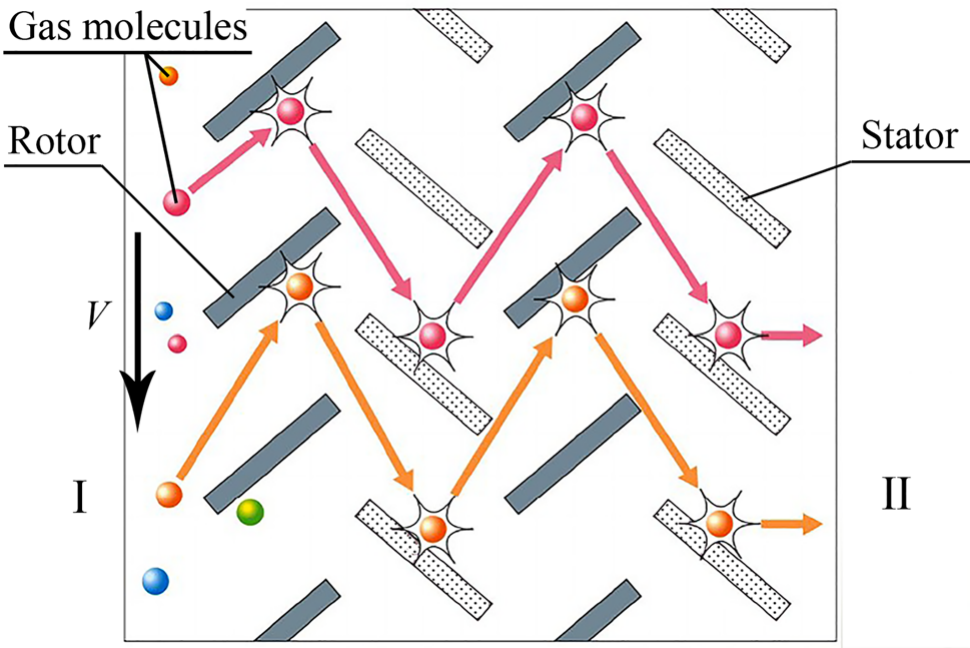
Schematic diagram of the working principle of the turbo molecular pump.

A turbomolecular pump (TMP) is a type of traction molecular pump that consists of a rotor composed of slotted disks or turbine blades and a stator. The rotor rotates between the corresponding slotted disks on the stator. The linear velocity of the turbine rotor is on the same order of magnitude as the velocity of gas molecules. This rotor operates under high-vacuum and ultrahigh-vacuum conditions, where the gas flow is in a free molecular flow state. In Fig. 1, Region I represents low pressure, and Region II represents high pressure. When the turbine blades rotate at a high linear velocity V, the gas molecules colliding with them move at high speeds. After diffuse or elastic reflection, the molecules move towards the stator blades, collide with them, and fly towards the rotor blades of the next stage. In this manner, the continuous rotation of the rotor blades causes gas molecules to continuously flow from the low-pressure region to the high-pressure region. Therefore, an extraction effect is generated, and a low working pressure inside the container is maintained. The working principle of the turbomolecular pump is shown in Fig. 1.

According to the working principle of the TMP, the structure of a multistage combined blade row (rotor–stator–rotor–stator rows) is partitioned into different regions, and an actual three-dimensional geometric model is established instead of a two-dimensional structure or a single-stage or two-stage blade row structure model. The established model has more than four combined blade rows, and the rotor–stator–rotor–stator row is intended to show the partial blade row structure of the TMP. The last stage can be set as either a stator blade row or a rotor blade row. The blade root angle, blade tip angle, blade root thickness and blade tip thickness parameters are introduced into the blade equations. The gap between the rotor and stator is considered. The blade equations of each blade are partitioned in the coordinate system. While tracking gas molecules, the algorithm relies on the shortest flight time to determine the wall boundary for collision and counts the number of collisions between gas molecules and the wall boundary. Thus, a new multistage combined blade row performance prediction algorithm according to the gas molecule flight time as the criterion is proposed.

### Setting the boundary conditions

**Figure 2:**
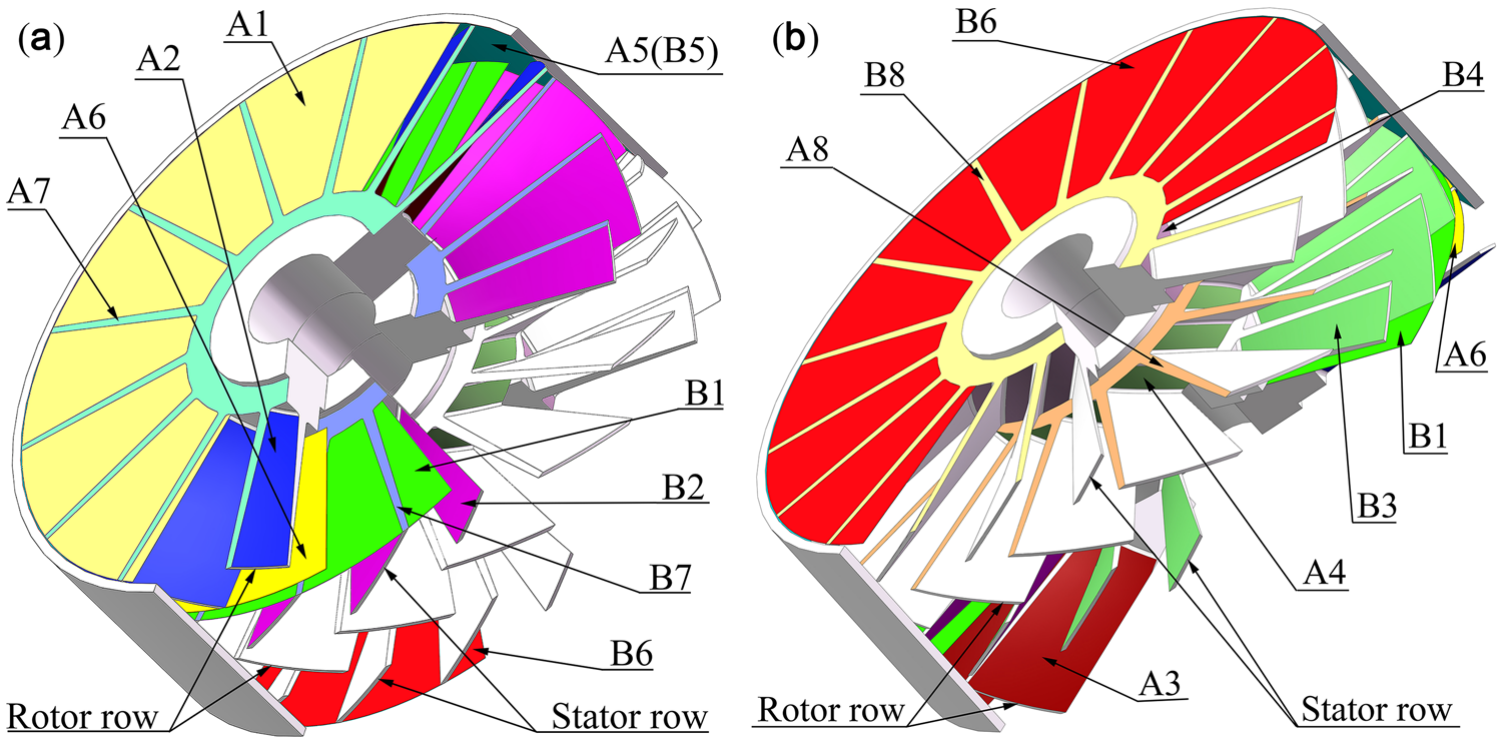
Rotor rows and stator rows partitioned into 8 boundaries. (**a**) and (**b**) come from different observation orientations of I and II, respectively.

The solution boundary of the rotor rows is partitioned into 8 boundaries, which are denoted as A1–A8, as shown in Fig. 2. Similarly, the stator row has 8 regions (B1–B8), and it considers the reflection effects of adjacent turbine blade rows. Therefore, the solution region of the multistage combined blade row is the cylindrical region between A1 and B8.

A2, A3, A4, A7 and A8 are motion boundaries. The velocity of gas molecules from the moving boundaries combines the rotor blade row tangential velocities and diffuse reflection velocities. B2, B3, B4, B5, B7, B8 and A5 are solid boundaries. Gas molecules from the solid boundaries only have diffuse reflection velocities. A1 and B1 are the inlet boundaries of each blade row. A6 and B6 are the outlet boundaries of each blade row.

**Figure 3:**
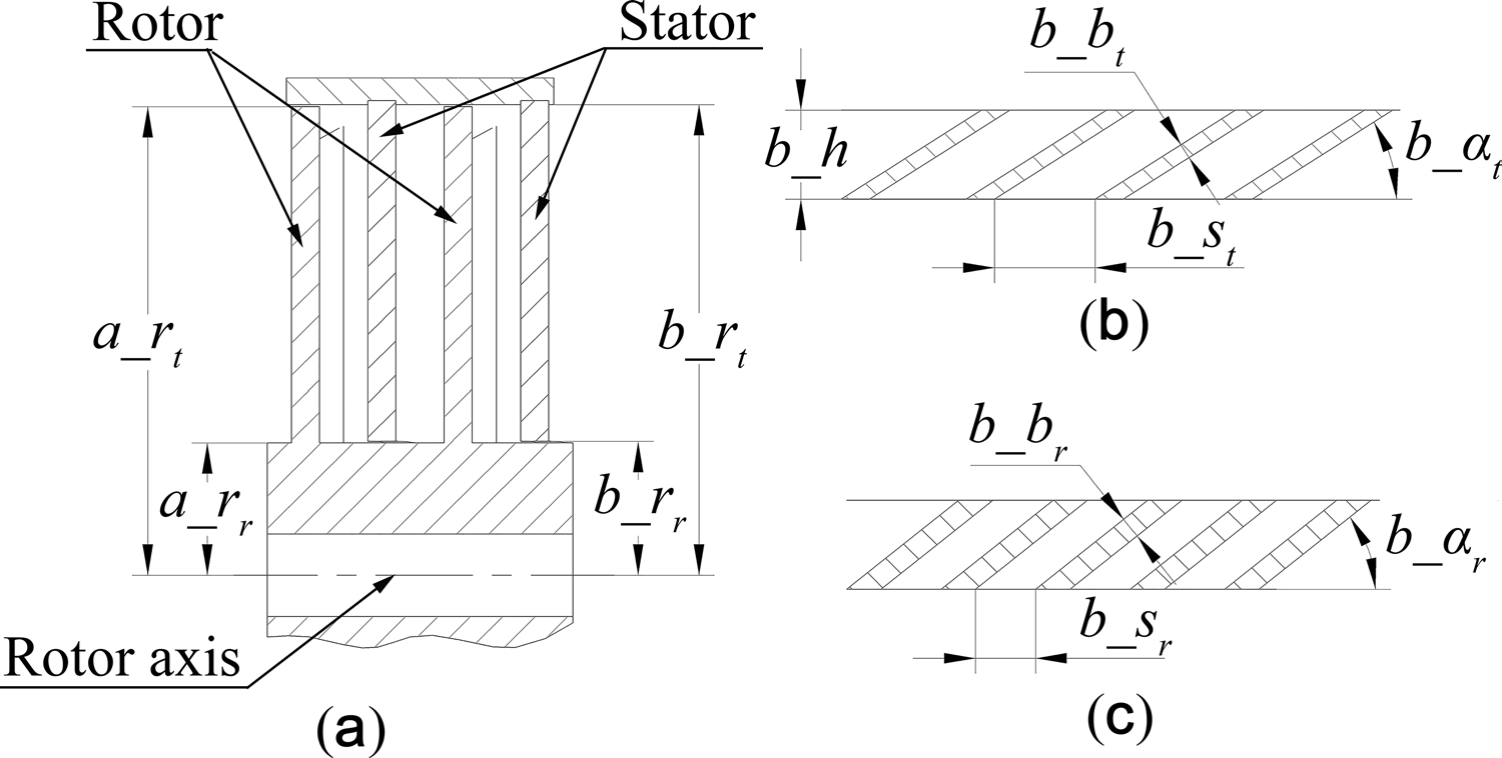
Blade cross-sectional view: (**a**) rotor–stator–rotor–stator, (**b**) stator blade tip and (**c**) stator blade root.

The diagrammatic cross-sections of the rotor–stator–rotor–stator and stator blade rows are shown in Fig. 3. The involved geometric parameters are labelled. Since the rotor blade rows and stator blade rows have similar geometric structures, a diagrammatic cross section of the rotor blade row is not provided.

### Blade equation

#### Building the Cartesian coordinate system

**Figure 4:**
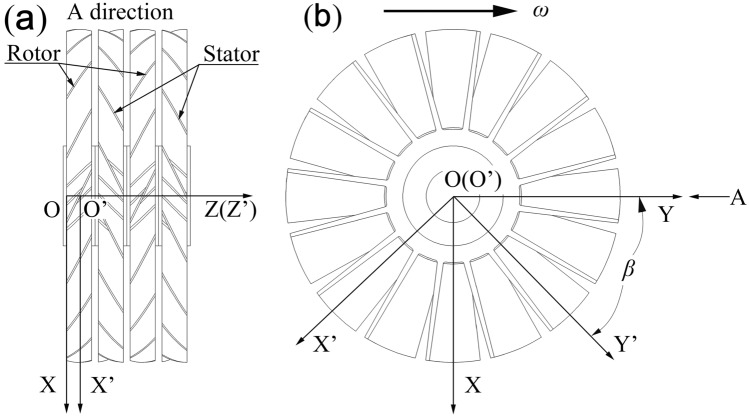
Relative position between the moving and static coordinate systems in the rotor–stator–rotor–stator blade rows: (**a**) A-direction view and (**b**) top plan view.

First, the coordinate system (OXYZ) is built for the multistage combined blade row, which is static relative to the stator row. The original point O is located on the centreline of the casing. The positive direction of the Z-axis is the pumping direction. The regions formed by the X-axis and the Y-axis coincide with the A1 region. The relative positions between the moving and static coordinate systems in the rotor–stator–rotor–stator blade row are shown in Fig. 4.

Later, a moving coordinate system O’X’Y’Z’ is created, and it is static relative to the rotor blade rows. The Y’-axis is the blade centreline. The original point O’ is located at the centre of the rotor blade rows. The Z’-axis positive direction is the pumping direction, as shown in Fig. 4.

Therefore, the coordinate system conversion relation under moving and static coordinate systems is given by Eqs. (1) and (2).

1 $$  \left\{ {\begin{array}{*{20}l}    {x^{\prime }  = x{\text{cos}}\beta  - y{\text{sin}}\beta } \hfill & {} \hfill  \\    {y^{\prime }  = y{\text{cos}}\beta  + x{\text{sin}}\beta ,} \hfill & {a\_r_{r}^{2}  < y^{2}  + x^{2}  < a\_r_{t}^{2} } \hfill  \\    {z^{\prime }  = z - a\_h/2} \hfill & {0 \le z \le a\_h_{1}  + b\_h_{1}  + a\_h_{2}  + b\_h_{2}  + 3\delta } \hfill  \\   \end{array} } \right.  $$

2 $$  {\text{or}}\;\left\{ {\begin{array}{*{20}l}    {x = x^{\prime } {\text{cos}}\beta  + y^{\prime } {\text{sin}}\beta } \hfill & {} \hfill  \\    {y = y^{\prime } {\text{cos}}\beta  - x^{\prime } {\text{sin}}\beta ,} \hfill & {a\_r_{r}^{2}  < y^{{\prime 2}}  + x^{{\prime 2}}  < a\_r_{t}^{2} } \hfill  \\    {z = z^{\prime }  + a\_h/2} \hfill & { - a\_h_{1} /2 \le z^{\prime }  \le a\_h_{1} /2 + b\_h_{1}  + a\_h_{2}  + b\_h_{2} } \hfill  \\   \end{array} } \right.  $$

where $\beta { = }\omega \, t$; $\omega$ is the angular velocity of the turbine rotor blade rows and $t$ is the gas molecule flight time from the inlet to the outlet.

#### Solving blade equations

According to the geometric position relationship of the blade in Fig. 4, the equation for blade No. 0 in the rotor blade rows (namely, A2) under the moving coordinate system can be obtained by Eq. (3).

3 $$  \left\{ {\begin{array}{*{20}l}    { - \frac{{a\_h}}{{2{\text{tan}}a\_\alpha }} - \frac{{a\_b}}{{2\sin a\_\alpha }} \le x^{\prime }  \le \frac{{a\_h}}{{2{\text{tan}}\,a\_\alpha }} - \frac{{a\_b}}{{2\sin a\_\alpha }}} \hfill  \\    {a\_r_{r}  \le r \le a\_r_{t} ,{\text{    }}r = x^{{\prime 2}}  + y^{{\prime 2}} } \hfill  \\    {z^{\prime }  =  - \left( {x^{\prime }  + \frac{{a\_b}}{{2\sin a\_\alpha }}} \right){\text{tan}}\,a\_\alpha } \hfill  \\   \end{array} } \right.  $$

The inclination angle $a\_\alpha$ and blade thickness $a\_b$ in Eq. (3) are functions of change. This idea is first proposed, and the detailed solution process is provided in the following text.

Under the static coordinate system, combined with Eq. (1), the A2 blade No. $a\_m$ equation in rotor blade rows is expressed by Eq. (4) at running time $t$ and angular velocity $\omega$. The detailed solving process is given in the Supplementary Materials.

4 $$ x{\text{cos}}\left( {\frac{2\pi a\_m}{{a\_n}} + \omega t} \right) - y{\text{sin}}\left( {\frac{2\pi a\_m}{{a\_n}} + \omega t} \right) + \left( {z - \frac{a\_h}{2}} \right){\text{cot}}a\_\alpha + \frac{a\_b}{{2\sin a\_\alpha }} = 0 $$

where $a\_m$ is the number of blades and is solved by Eq. (5).

5 $$ a\_m = {\text{Int}}\left( {\frac{\theta }{2\pi } \cdot a\_n} \right),0 \le \theta < 2\pi $$

$\theta$ depends on the position of the gas molecules, as shown in Eq. (6).

6 $$  \theta  = \left\{ {\begin{array}{*{20}c}    {{\text{arccos}}\left( {\frac{y}{{\sqrt {x^{2}  + y^{2} } }}} \right)} & {a\_r_{r}  < y < a\_r_{t} \;x \ge 0}  \\    {{\text{2}}\pi {\text{ - arccos}}\left( {\frac{y}{{\sqrt {x^{2}  + y^{2} } }}} \right)} & {a\_r_{r}  < y < a\_r_{t} \;x < 0}  \\   \end{array} } \right.  $$

Similarly, the A3 blade equation in rotor blade rows is expressed by Eq. (7).

7 $$ x{\text{cos}}\left( {\frac{2\pi a\_m}{{a\_n}} + \omega t} \right) - y{\text{sin}}\left( {\frac{2\pi a\_m}{{a\_n}} + \omega t} \right) + \left( {z - \frac{a\_h}{2}} \right){\text{cot}}a\_\alpha - \frac{a\_b}{{2\sin a\_\alpha }} = 0 $$

For the blade equations (B2 or B3) in the stator blade rows, the derivation process is the same as that for the A2 and A3 blade equations. The stator blades have no rotational speed. Therefore, the blade equations of B2 and B3 are solved by Eqs. (8) and (9), respectively.

8 $$ - x{\text{cos}}\left( {\frac{2\pi b\_m}{{b\_n}}} \right) + y{\text{sin}}\left( {\frac{2\pi b\_m}{{b\_n}}} \right) + \left( {z - \frac{b\_h}{2}} \right){\text{cot}}b\_\alpha + \frac{b\_b}{{2\sin b\_\alpha }} = 0 $$

9 $$ - x{\text{cos}}\left( {\frac{2\pi b\_m}{{b\_n}}} \right) + y{\text{sin}}\left( {\frac{2\pi b\_m}{{b\_n}}} \right) + \left( {z - \frac{b\_h}{2}} \right){\text{cot}}b\_\alpha - \frac{b\_b}{{2\sin b\_\alpha }} = 0 $$

The above blade equations are derived in an independent coordinate system. However, in the actual simulation and calculation algorithm, the change in the coordinate in the Z-axis direction is updated in real time.

In addition, the blade root angle, blade tip angle, blade root thickness and blade tip thickness parameters are considered in the blade equations, which was not done in previous research. Thus, the rotor blade angle $a\_\alpha$ and stator blade angle $b\_\alpha$ are represented by Eqs. (10) and (11), respectively. The rotor blade thickness $a\_b$ and stator blade thickness $b\_b$ are given by Eqs. (12) and (13), respectively.

10 $$ a\_\alpha = \frac{{a\_\alpha_{t} - a\_\alpha_{r} }}{{a\_r_{t} - a\_r_{r} }}y_{a}^{\prime } { + }\frac{{a\_r_{t} \cdot a\_\alpha_{r} - a\_r_{r} \cdot a\_\alpha_{t} }}{{a\_r_{t} - a\_r_{r} }} $$

11 $$ b\_\alpha = \frac{{b\_\alpha_{t} - b\_\alpha_{r} }}{{b\_r_{t} - b\_r_{r} }}y_{b}^{\prime } { + }\frac{{b\_r_{t} \cdot b\_\alpha_{r} - b\_r_{r} \cdot b\_\alpha_{t} }}{{b\_r_{t} - b\_r_{r} }} $$

12 $$ a\_b = \frac{{a\_b_{t} - a\_b_{r} }}{{a\_r_{t} - a\_r_{r} }}y_{a}^{\prime } { + }\frac{{a\_r_{t} \cdot a\_b_{r} - a\_r_{r} \cdot a\_b_{t} }}{{a\_r_{t} - a\_r_{r} }} $$

13 $$ b\_b = \frac{{b\_b_{t} - b\_b_{r} }}{{b\_r_{t} - b\_r_{r} }}y_{b}^{\prime } { + }\frac{{b\_r_{t} \cdot b\_b_{r} - b\_r_{r} \cdot b\_b_{t} }}{{b\_r_{t} - b\_r_{r} }} $$

where $y_{a}^{\prime }$ and $y_{b}^{\prime }$ are solved by Eqs. (14) and (15), respectively.

14 $$ y_{a}^{\prime } = y{\text{cos}}\left( {\frac{2\pi a\_m}{{a\_n}} + \omega t} \right) + x{\text{sin}}\left( {\frac{2\pi a\_m}{{a\_n}} + \omega t} \right) $$

15 $$ y_{b}^{\prime } = y{\text{cos}}\left( {\frac{2\pi b\_m}{{b\_n}}} \right) + x{\text{sin}}\left( {\frac{2\pi b\_m}{{b\_n}}} \right) $$

#### Solution of the gas molecule flight time

Supposing that the gas molecules from one boundary to another boundary are straight lines, each molecular motion equation can be obtained by Eq. (16).

16 $$ \left\{ \begin{gathered} x_{i} = x_{j} + v_{xj} \cdot t \hfill \\ y_{i} = y_{j} + v_{yj} \cdot t \hfill \\ z_{i} = z_{j} + v_{zj} \cdot t \hfill \\ \end{gathered} \right. $$

In Eq. (16), $x_{j}$ is the x-coordinate (initial position) in region $j$, $v_{xj}$ is the reflection velocity (initial position) in the x-direction of region $j$, $x_{i}$ is the x-coordinate (end position) in region $i$, and $j$ and $i$ are between 1 and 8, but $j$ and $i$ are not equal. The equations for solving the gas molecule flight time $t$ from one boundary to another boundary are obtained, as shown in Table 1. The gas molecules can only reach the remaining 7 regions because it is impossible to return to the same region.

**Table 1:** Equations for the gas molecule flight time $t$ from one boundary to another boundary.

Gas molecules in rotor rows	Solving equations, $j \in \left[ {1,8} \right]$	Gas molecules in stator row	Solving equations, $j \in \left[ {1,8} \right]$
$at_{j1}$	$z_{j} /v_{zj}$	$bt_{j1}$	$z_{j} /v_{zj}$
$at_{j2}$	Combined Eq. (16) with Eq. (4), gas molecule flight time $t$ is solved	$bt_{j2}$	Combined Eq. (16) with Eq. (8), gas molecule flight time $t$ is solved
$at_{j3}$	Combined Eq. (16) with Eq. (7), gas molecule flight time $t$ is solved	$bt_{j3}$	Combined Eq. (16) with Eq. (9), gas molecule flight time $t$ is solved
$at_{j4}$	The rotor body surface Equation (A4) is given as $x^{2} + y^{2} = a\_r_{r}^{2}$. Combined Eq. (16), gas molecule flight time $t$ is obtained	$bt_{j4}$	The stator body surface Equation (B4) is given as $x^{2} + y^{2} = b\_r_{r}^{2}$. Combined Eq. (16), gas molecule flight time $t$ is obtained
$at_{j5}$	The casing surface Equation (A5) is given as $x^{2} + y^{2} = a\_r_{t}^{2}$. Combined Eq. (16), the gas molecule flight time $t$ is solved	$bt_{j5}$	The casing surface Equation (B5) is given as $x^{2} + y^{2} = b\_r_{t}^{2}$. Combined Eq. (16), the gas molecule flight time $t$ is solved
$at_{j6}$	$\left( {a\_h - z_{j} } \right)/v_{zj}$	$bt_{j6}$	$\left( {b\_h - z_{j} } \right)/v_{zj}$
$at_{j7}$	$z_{j} /v_{zj}$	$bt_{j7}$	$z_{j} /v_{zj}$
$at_{j8}$	$\left( {a\_h - z_{j} } \right)/v_{zj}$	$bt_{j8}$	$\left( {b\_h - z_{j} } \right)/v_{zj}$

Annotation: In the actual simulation and calculation algorithm, the change in the coordinate in the Z-axis direction is updated in real time.

## Research method and basic theory

### Research method

The Monte Carlo method^20–22^ has been widely applied in many situations. In our previous research^19,23,24^, the inlet structure and pumping mechanism of TMPs are studied via this method. To accurately trace the gas molecule flight track in the multistage combined blade row, a particle replaces a molecule. In this manner, all the gas molecules are replaced with test particles. This research method is called the TPMC method^25^. In this work, the TPMC method is used to study the pumping performance of a TSBR, and a flow field analysis of the VSCBR is performed. This analysis can simulate the movement of gas molecules very well.

### Basic theory

The positive transmission probability $\sum_{12}$ is the probability from upstream to downstream. The reverse transmission probability $\sum_{21}$ is the probability from downstream to upstream. $\sum_{12}$ and $\sum_{21}$ depend on the number of collisions between gas molecules and the outlet region. The maximum compression ratio ($K_{\max }$) and the maximum pumping speed factor ($Q_{\max }$) of the multistage combined blade rows are expressed by Eqs. (17) and (18). $S_{1}$ and $S_{2}$ are the effective areas of the inlet blade rows and outlet blade row, respectively, which increases the accuracy of the calculated results of the maximum pumping speed factor and maximum compression ratio. The values of *S*_1_ and *S*_2_ have not been considered in previous research^19^. The maximum pumping speed factor and maximum compression ratio are two important performance parameters of the TMP^1^.

17 $$ K_{\max } = \frac{{S_{1} \cdot \sum_{12} }}{{S_{2} \cdot \sum_{21} }} $$

18 $$ Q_{\max } = \sum_{12} - \frac{{S_{2} }}{{S_{1} }}\sum_{21} $$

When calculating the positive transmission probability, the gas molecules in the A1 region have a uniform distribution. The initial position of each molecule can be represented by a random number ($R_{f}$), as shown in Eq. (19). The velocity distribution of gas molecules obeys the Maxwellian velocity distribution. The initial velocity of each molecule is randomly created by Eq. (20).

19 $$ \left\{ \begin{gathered} x_{{_{0} }} = \sqrt {\left[ {a\_r_{r}^{2} + R_{f} \left( {a\_r_{t}^{2} - a\_r_{r}^{2} } \right)} \right]} \hfill \\ y_{{_{0} }} = \sqrt {\left[ {a\_r_{r}^{2} + R_{f} \left( {a\_r_{t}^{2} - a\_r_{r}^{2} } \right)} \right]} \hfill \\ z_{{_{0} }} = 0 \hfill \\ \end{gathered} \right.,0 < R_{f} < 1 $$

20 $$ \left\{ \begin{gathered} v_{x0} = \sqrt { - \ln R_{f} } V_{{{\text{mp}}}} {\text{sin}}\left( {2\pi R_{f} } \right) \hfill \\ v_{y0} = \sqrt { - \ln R_{f} } V_{{{\text{mp}}}} {\text{cos}}\left( {2\pi R_{f} } \right) \hfill \\ v_{z0} = \sqrt { - \ln R_{f} } V_{{{\text{mp}}}} \hfill \\ \end{gathered} \right. $$

The most probable velocity $V_{{{\text{mp}}}}$ is equal to $\sqrt {2RT/M}$. When calculating the reverse transmission probability, the gas molecules are randomly created in the B6 region. The initial position $z_{0}$ and velocity $v_{z0}$ need to be replaced with $z_{0} = a\_h_{1} + a\_h_{2} + b\_h_{1} + b\_h_{2} + 3\delta$ and $v_{z0} = - \sqrt { - \ln R_{f} } V_{{{\text{mp}}}}$, respectively. The other involved parameters remain the same.

The diffusive collision model is employed when the gas molecules collide with the solid boundaries. The shortest flight time from one boundary to another boundary is the criterion for determining the collision position of gas molecules with solid boundaries. Depending on the collision position coordinates with the solid boundaries, the number of gas molecules reaching each boundary is recorded.

## Experimental testing and verification

**Figure 5:**
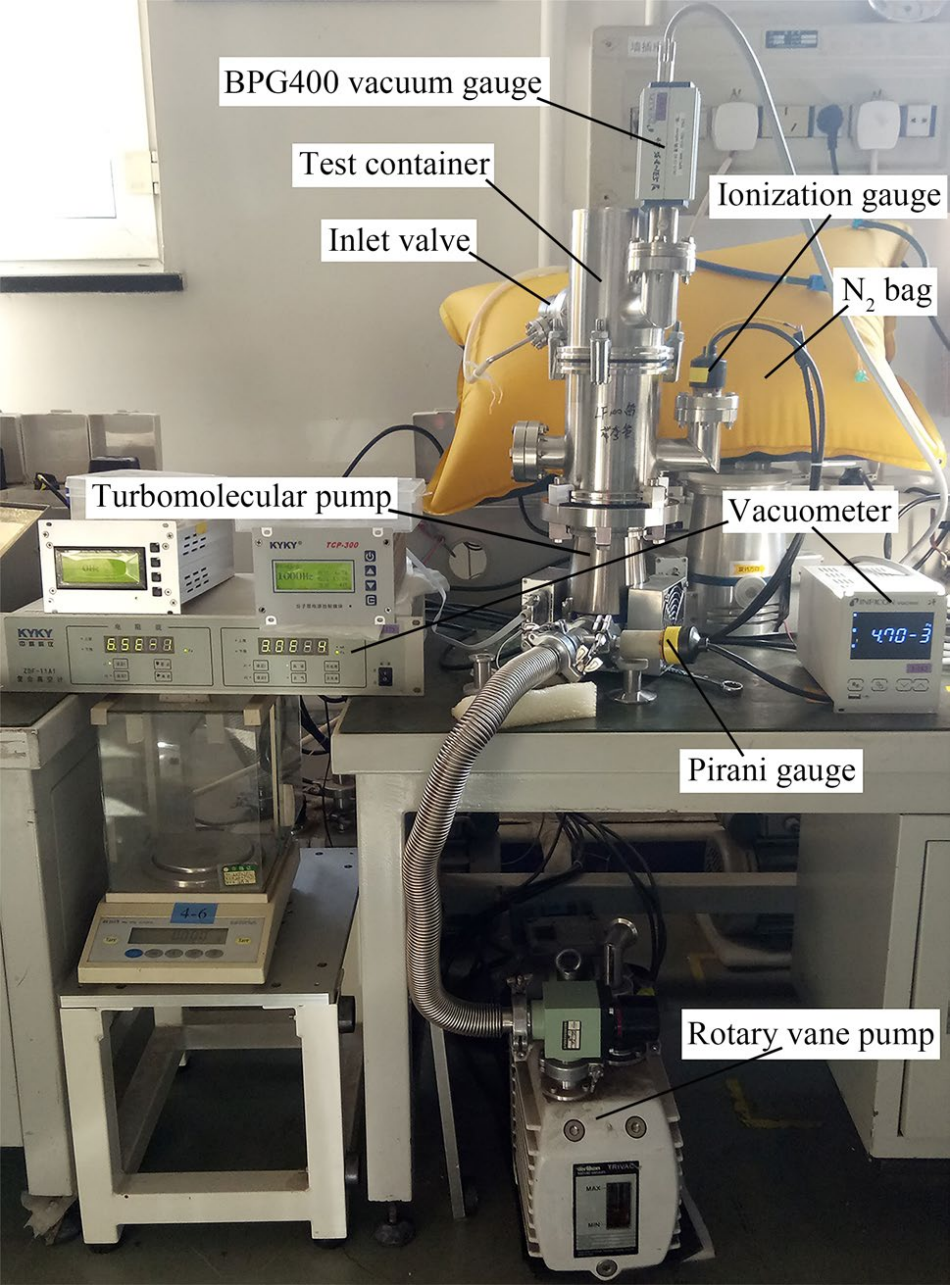
Pumping performance of the TMP.

To verify the feasibility and accuracy of the proposed simulation calculation algorithm and established geometric structure model, the FF-63/80 TMP (produced by *KYKY Technology Co*., *Ltd*.) is selected for experimental testing and comparative research. The pumping performance measurement device is a standard test system for TMP, as shown in Fig. 5.

Considering the issue of intellectual property protection for the FF-63/80 TMP product, only the geometric parameters of the first 8 stages of turbine combined blade rows are provided, as shown in Table 2. The operating conditions of this experiment are as follows: the rotational speed of the TMP is 60,000 rpm, the room temperature is 20 $^\circ {\text{C}}$, the humidity is 18%, and the gas medium is nitrogen.

**Table 2:** Geometric parameters of the first 8 stages of FF-63/80 TMP.

Stage No.	Blades root diameter (mm)	Blades tip diameter (mm)	Blades height (mm)	Blades thickness (mm)	Number of blades teeth	Blades angle (°)
1-rotor	39	68	5	1	16	40
2-stator	43	72	4	0.8	28	35
3-rotor	44	68	3	0.6	26	33
4-stator	47	72	2.5	0.6	30	28
5-rotor	47	68	2.5	0.6	24	26
6-stator	50	72	2.5	0.5	20	16
7-rotor	50	68	2.5	0.4	22	18
8-stator	50	72	2.5	0.5	20	16

The detailed measurement devices and selected types have been introduced in previous research^24^ and will not be described here. The error ranges of the INFCON BPG400 gauge, ZJ-27 hot cathode gauge and ZJ-52T gauge involved in the measurement devices can meet the measurement error requirements of this experimental test.

For the measurement methods and procedures, the current international standard ISO 21,360–4:2018 (Vacuum technology—Standard methods for measuring vacuum-pump performance—Part 4: Turbomolecular vacuum pumps) is selected instead of the Chinese national standard GB/T 7774–2007 (equivalent to international standard ISO 5302:2003). To increase the accuracy of the experimental results and reduce the measurement error, when the experimental procedure is strictly performed, each pressure measurement point is maintained for more than 5 min, the fluctuation error of the vacuum gauge is within 5%, and the measurement value is effective; otherwise, the measurement is performed again. Moreover, each pressure measurement point is measured multiple times, and the average is taken.

The simulation calculation is carried out according to the geometric model and performance prediction algorithm of the VSCBR set up above. The geometric structural parameters for the simulation calculations are derived from Table 3. During the implementation process, the blade root angle is equal to the blade tip angle, and the blade root thickness is equal to the blade tip thickness. $y_{a}^{\prime }$ and $y_{b}^{\prime }$ in Eqs. (14) and (15) are set to 0. Thus, the newly proposed performance prediction algorithm can predict the pumping performance of the TSBR and VSCBR.

**Table 3:** Geometric parameters of the rotor–stator–rotor–stator blade row^26^.

Stage	Blades root radius (mm)	Blades tip radius (mm)	Blades height (mm)	Blades thickness (mm)	Number of blades teeth	Blades angle (°)
First stage rotor	22	33.5	6	0.8	16	40
Second stage stator	22	33.5	3	0.3	26	30
Third stage rotor	26	33.5	2	0.8	24	30
Fourth stage stator	26	33.5	3	0.3	30	26

**Figure 6:**
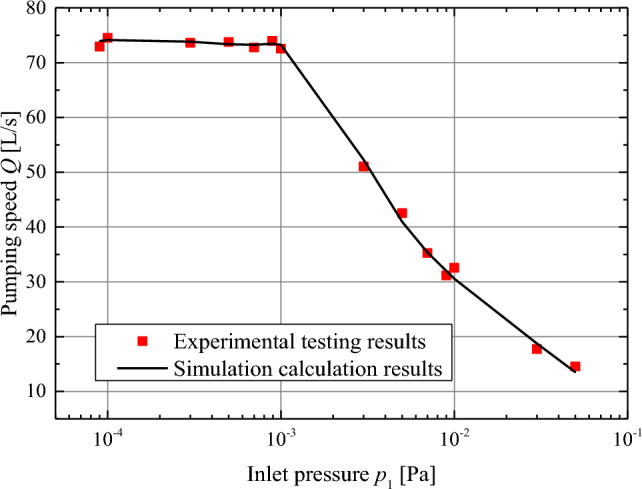
Experimental testing and simulation calculation results of TMP for pumping speed.

Figure 6 shows the experimental test and simulation results for the pumping speed. Each simulation calculation result is obtained based on simulating many molecules, and each experimental test result is measured through strict adherence to the international standard ISO 21,360–4:2018. It is not difficult to observe that the simulation calculation results are almost consistent with the experimental testing, which is sufficient to prove the feasibility and accuracy of the proposed performance prediction algorithm and geometric model for TMP.

**Figure 7:**
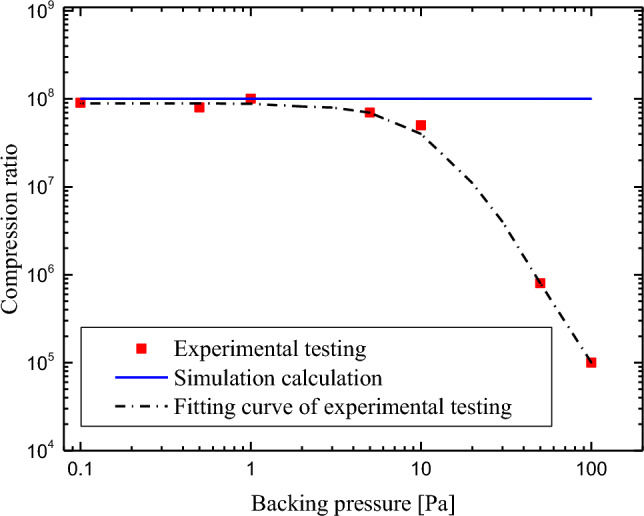
Experimental testing and simulation calculation results of the TMP for the compression ratio.

To verify the feasibility and accuracy of the newly proposed performance prediction algorithm, another important parameter that can reflect the performance of the TMP, the compression ratio, is selected for experimental testing and simulation calculations. According to Fig. 7, the simulation calculation results remain unchanged with the backing pressure, with a value of 1.01 × 10^8^, because it is calculated based on Eq. (17). Moreover, this value is greater than the fitting value of the experimental test (approximately 8.97 × 10^7^). According to the analysis, the reason for this phenomenon is residual gas leakage from measuring devices, such as test containers and vacuum gauges. Leakage increases the pressure limit established at the inlet of the TMP, which in turn causes the compression ratio measured in the experiment to be lower than the simulated value. However, the errors caused by these factors are within the allowable range of the ISO 21,360–4:2018 measurement standard. This finding verifies the accuracy of the simulation calculation algorithm.

When the backing pressure (i.e., the outlet pressure of the TMP) is greater than 5 Pa, the measured compression ratio decreases, as shown in Fig. 7. This result arises because the gas at this time is no longer in a free molecular flow state, and the TMP is no longer in a normal working state. Therefore, TMP is always in a free molecular flow state during operation.

## Results and discussion

### Pumping performance

In this section, the variation in the pumping performance as a function of the blade velocity ratio ($c = \pi \omega (a\_r_{r} + a\_r_{t} )/V_{{{\text{mp}}}}$) is investigated via a designed simulation. The structural parameters of the rotor–stator–rotor–stator blade row in Table 3 are used to research the pumping performance and are obtained from the F-63/55 TMP^26^. These parameters are obtained because the first four stages of combined blade row have an outstanding impact on the pumping speed.

**Figure 8:**
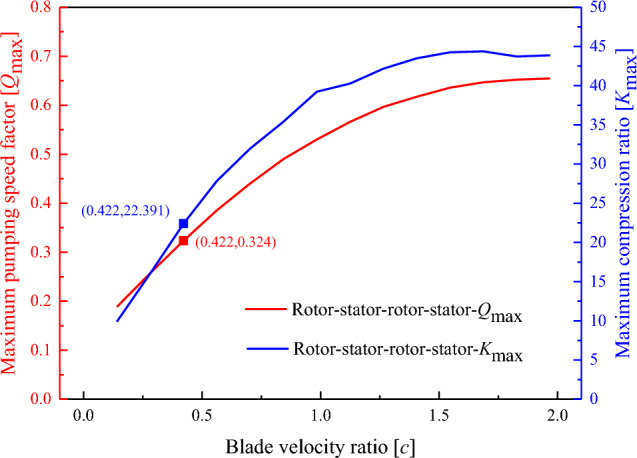
Variation in the pumping performance of the rotor–stator–rotor–stator system with the blade velocity ratio.

When calculating the pumping performance of the TSBR, the blade thickness and blade angle values are fixed. When $c < 1$, the maximum pumping speed factor $Q_{\max }$ and maximum compression ratio $K_{\max }$ increase with increasing blade velocity ratio, as shown in Fig. 8. This trend is consistent with the actual situation in which the pumping speed and compression ratio increase with increasing blade rotational speed. However, when $c > 1$, the increase in the maximum pumping speed factor and maximum compression ratio gradually stabilizes. This result is different from the previous basic theory^27^, in which the relationship between the maximum compression ratio and blade velocity ratio is an exponential function. With the TSBR structure, the gas molecules reach saturation when the TMP is high. Therefore, it is unreasonable to increase the pumping speed and compression ratio by simply increasing the rotational speed under an unchanging turbine blade structure.

We hope that the pumping speed and compression ratio can greatly increase after the blade rotational speed increases. Thus, it is very important to improve the pumping performance by optimizing the TSBR. This study is consistent with current simulation calculation demands and development trends for high-speed TMPs.

To compare the pumping performance of the optimized structural parameters based on the new performance prediction algorithm with that of the TSBR structural model, when the blade rotational speed of the TMP is 60,000 rpm, the simulation calculation values of *Q*_max_ and *K*_max_ are 0.324 and 22.391, respectively, as shown in Fig. 8.

### Optimization of the TSBR structure

Based on the VSCBR geometric structure model, the response surface methodology is used to optimize the TSBR structural parameters of the first four-stage combined blade row, which have a significant impact on the pumping speed. The original structural parameters are shown in Table 3. The structural and operational parameters of the original pump chamber and motor are not changed. By gradually optimizing the blade root thickness, blade tip thickness, blade root angle, and blade tip angle, the aim of improving the pumping performance of TMPs can be achieved.

On the basis of single-factor simulations, the maximum pumping speed factor (*Y*) is taken as the response value, and the four factors of the first-stage rotor blade row, namely, the blade root thickness (*X*_1_), blade tip thickness (*X*_2_), blade root angle (*X*_3_), and blade tip angle (*X*_4_), are taken as the investigation factors. The Box‒Behnken simulation factors and levels are shown in Table 4. The simulation results and analysis are shown in Table 5.

**Table 4:** Box‒Behnken simulation factors and levels of the first-stage rotor blade row.

Factors	− 1	0	1
*X*_1_ (mm)	0.4	0.8	1.2
*X*_2_ (mm)	0.4	0.8	1.2
*X*_3_ (°)	35	40	45
*X*_4_ (°)	35	40	45

**Table 5:** Box‒Behnken simulation results and analyses of the first-stage rotor blade rows.

No	*X*_1_	*X*_2_	*X*_3_	*X*_4_	*Y*	No	*X*_1_	*X*_2_	*X*_3_	*X*_4_	*Y*
1	0.8	0.4	45	40	0.3301	16	1.2	0.8	40	35	0.3218
2	0.4	0.8	40	35	0.3139	17	0.4	0.4	40	40	0.3230
3	1.2	0.8	45	40	0.3372	18	0.4	1.2	40	40	0.3200
4	1.2	0.8	35	40	0.3251	19	0.8	1.2	40	45	0.3235
5	0.8	0.8	40	40	0.3281	20	0.4	0.8	35	40	0.3069
6	0.8	0.8	35	45	0.3135	21	0.4	0.8	45	40	0.3261
7	0.8	0.4	40	35	0.3202	22	0.8	0.8	40	40	0.3285
8	0.8	1.2	40	35	0.3161	23	0.4	0.8	40	45	0.3176
9	1.2	0.8	40	45	0.3226	24	0.8	0.8	45	45	0.3348
10	0.8	0.4	35	40	0.3187	25	0.8	0.8	40	40	0.3287
11	0.8	0.8	40	40	0.3276	26	0.8	1.2	35	40	0.3076
12	0.8	0.8	45	35	0.3235	27	0.8	0.8	40	40	0.3270
13	0.8	0.4	40	45	0.3332	28	1.2	0.4	40	40	0.3372
14	1.2	1.2	40	40	0.3329	29	0.8	0.8	35	35	0.3112
15	0.8	1.2	45	40	0.3299						

The simulation results in Table 5 are fitted by using Design Expert V8.0.6 software to obtain the regression equation, as shown in Eq. (21).

21 $$ \begin{aligned} Y_{1} & = - 0.1267 + 0.0144X_{1} - 0.0611X_{2} + 7.9877 \times 10^{ - 3} X_{3} + 0.0132X_{4} \\ & \quad + 1.3581 \times 10^{ - 3} X_{2} X_{3} + 9.0465 \times 10^{ - 5} X_{3} X_{4} - 1.3809 \times 10^{ - 4} \, X_{3}^{2} - 2.0216 \times 10^{ - 4} \, X_{4}^{2} \\ \end{aligned} $$

Stepwise regression is performed for the obtained regression equation to determine the optimal structural parameters of the first-stage rotor blade rows, including a blade root thickness of 1.20 mm, a blade tip thickness of 1.16 mm, a blade root angle of 45.00°, and a blade top angle of 42.73°. Based on the optimal structural parameters of the first-stage rotor row, the original structural parameters are selected for the other three-stage parameters. At this time, the maximum pumping speed factor prediction value of turbine combined blade rows is 0.3392 according to the regression equation. The simulated value for the maximum pumping speed factor is 0.3385 according to the VSCBR simulation calculation program, with a deviation of only 0.21% from the predicted value of the regression equation. This finding confirms the accuracies of the regression equation and simulation calculation algorithm.

In structural optimization research, the gap between each stage is 0.5 mm, and the gap between the pump casing (A5 or B5) and the blade tip is 0.5 mm. The gas molecular mass is 29, the room temperature is 25 °C, and the blade rotational speed is 60,000 rpm. In the past, the blade rotational speed is relatively low, and a low blade rotational speed is reasonable. If the blade rotational speed is relatively high, the optimal TSBR structure parameters are different.

Next, by using the same method and operating conditions, the TSBR structural parameters of the second-stage stator blade rows, third-stage rotor blade rows, and fourth-stage stator blade row are optimized based on the optimized structural parameters of the first-stage rotor blade rows. The optimized structural parameters and simulation results are shown in Table 6.

**Table 6:** Structural parameters and simulation results after TSBR structural optimization.

Stage No.	Blade thickness (mm)		Blade angle (°)		Maximum pumping speed factor	Pumping speed growth rate (%)
	Root thickness	Tip thickness	Root angle	Tip angle		
First-stage rotor blade rows	1.2	1.16	45.00	42.73	0.339	4.9
Second-stage stator blade rows	0.2	0.5	25.01	35.00	0.352	8.8
Third-stage rotor blade rows	0.8	0.4	30.00	25.00	0.366	13.1
Fourth-stage stator blade rows	0.1	0.3	25.00	30.00	0.382	18.2

**Figure 9:**
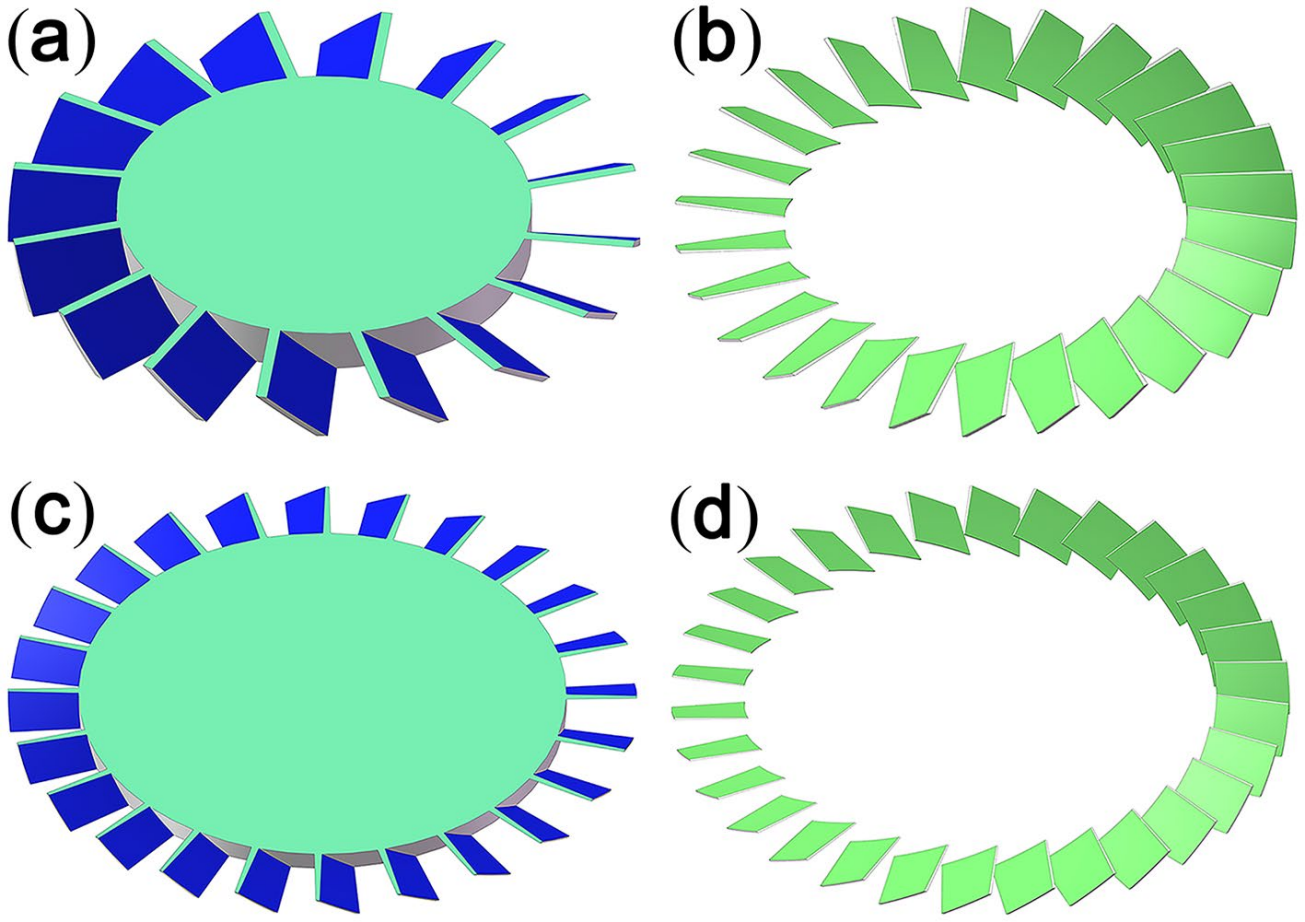
3D geometric structure modelling diagram of the optimized four-stage blades: (**a**) first-stage rotor blade rows, (**b**) second-stage stator blade rows, (**c**) third-stage rotor blade rows, and (**d**) fourth-stage stator blade rows.

According to the simulation results in Table 6, as the structural parameters of the four-stage combined blade rows continue to be optimized, the maximum pumping speed factor of the combined blade row gradually increases. The 3D geometric structure modelling diagram of the optimized four-stage blades is shown in Fig. 9. Compared with the original structural parameters (namely, the TSBR structure), the final optimized structure (namely, the VSCBR structure) increases the pumping speed by 18.2%. This result arises because the structural characteristics of the VSCBR are in agreement with the changes in molecular gas dynamics, indicating that the angle and thickness of the blades change with the blade radius. The VSCBR structure overcomes the limitations of improving the pumping performance caused by the TSBR structure. Therefore, a TMP with VSCBR structural characteristics is a future development trend. This trend allows TMPs to be widely used in scientific instruments (such as in portable helium mass spectrometry leak detectors) and industrial production processes (such as in the semiconductor and integrated circuit industries).

Through the response surface methodology prediction analysis mentioned above, the optimal solution results can be quickly found, and considerable simulation and calculation time can be saved. For the current development trend of high-rotational-speed TMPs, relevant experimental testing platforms based on VSCBR structural characteristics are under construction.

### Flow field analysis of the VSCBR structure

Based on the calculation algorithm of the VSCBR structure, flow field analysis is conducted on the optimized new combined blade row, the numbers of gas molecules in all regions are recorded by the written simulation calculation program, and the motion trajectories of all gas molecules are tracked by the TPMC method.

**Figure 10:**
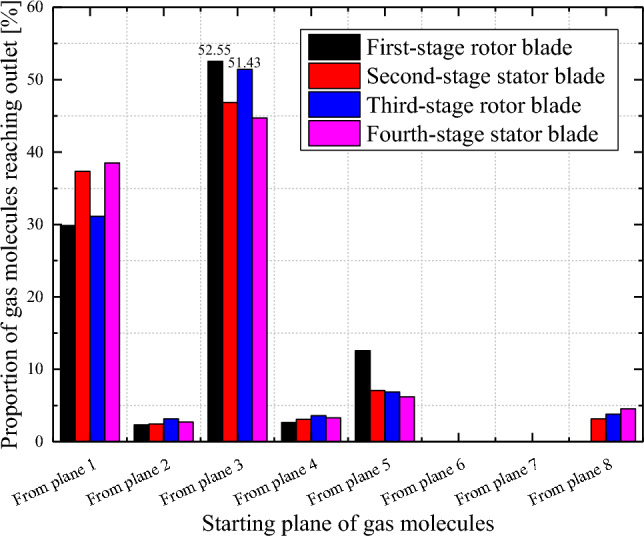
Proportion of gas molecules reaching the outlet of each blade stage.

Figure 10 shows the proportion of gas molecules starting from different regions to reach the outlet of each stage combined blade row. Gas molecules starting from the rear blades (Plane 3) easily arrive at the outlet (Plane 6) of the turbine blade rows. The proportions of gas molecules reaching the outlet in the first- and third-stage turbine blade rows reach 52.55% and 51.43%, respectively. Because only gas molecules that reach the outlet inside the turbine blade row can actually pump gas, the greater the number of molecules that can reach the rear blades (Plane 3), the more advantageous this system is for improving the pumping speed.

**Figure 11:**
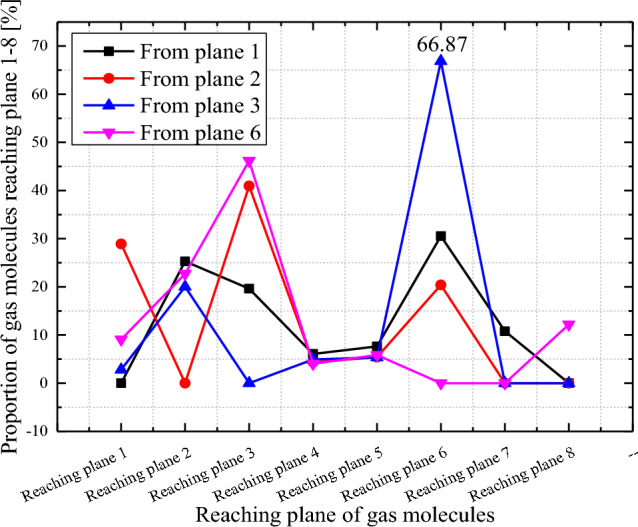
Proportions of gas molecules reaching A1–A8 in the third-stage rotor blade rows.

By taking third-stage rotor blade rows of the intermediate stage as an example for further analysis, the proportions of gas molecules reaching planes 1–8 from planes 1, 2, 3, and 6 are calculated, as shown in Fig. 11. It is obvious that the proportions of gas molecules from plane 3 to plane 6 are the highest, reaching 66.87%. This finding indicates that gas molecules reflected from the rear blades (Plane 3) of the rotor row are likely to reach the outlet (Plane 6) of the turbine blade row, which is favourable for pumping gas. By observing the proportion of gas molecules reaching plane 3, it is determined that the proportion of gas molecules returning from plane 6 and coming from plane 2 is relatively great, indicating that the VSCBR structure is beneficial for reducing the gas molecule backflow and increasing the pumping speed.

Through the flow field analysis shown in Figs. 10 and 11 above, it is found that when designing the structure of multistage combined blade rows, the blade inclination and blade thickness should be varied to increase the probability of collision between gas molecules and rear blades (plane 3), thereby allowing additional gas molecules to reach the outlet of multistage combined blade rows. Moreover, the direction for the structural design of high-performance TMPs under the situation of high rotational speed is noted. The turbine blade row needs to be designed into a VSCBR structure.

## Conclusions

A new performance prediction algorithm for a variable surface combined blade row structure that can increase pumping performance is proposed. The pumping performance of multistage combined blade rows is calculated based on the TPMC method. The simulation results and the experimental results are in excellent agreement. This agreement confirms the feasibility and accuracy of the new algorithm for performance prediction. Research on pumping performance indicates that when $c > 1$, the increase in *Q*_max_ and *K*_max_ gradually stabilizes. The traditional straight blade row structure limits the improvement in pumping performance. By taking the first four-stage combined blade rows that have a significant impact on the pumping speed as an example, optimization research on the traditional straight blade row is carried out by a specific simulation calculation program, and a set of optimal combined blade row structural parameters (namely, the variable surface combined blade row structure) is obtained. The pumping speed growth rate of the final optimized structural model increases by 18.2%. Moreover, using the optimized structural parameters as the model for further flow field analysis, the research shows that gas molecules that reach the rear blade (Plane 3) are likely to reach the outlet (Plane 6) of the TMP, and the proportion of reaching gas molecules exceeds 50%. When designing the structure of a multistage combined blade row, the blade inclination and blade thickness should be varied to increase the probability of collision between gas molecules and rear blades (plane 3), thereby improving the pumping performance of the TMP. This finding provides a direction for the structural optimization design of high-performance TMPs, and the structure of the turbine blade row needs to be designed into a variable surface combined blade row structure. In the next stage, experimental testing research is performed based on the proposed performance prediction algorithm of the variable surface combined blade row structure, verifying the results of the simulation calculations.

## Supplementary Information

Supplementary Information.

## Data Availability

All the data generated during the current study are included in this article.

## List of symbols

A1: Rotor inlet
A2: Rotor front blades
A3: Rotor rear blades
A4: Rotor body
A5 (B5): Casing
A6: Rotor outlet
A7: Rotor top surface
A8: Rotor bottom surface
$a\_r_{r}$: Rotor blade root radius
$a\_r_{t}$: Rotor blade tip radius
$a\_\alpha$: Rotor blade angle
$a\_\alpha_{r}$: Rotor blade root angle
$a\_\alpha_{t}$: Rotor blade tip angle
$a\_h$: Rotor blade height
$a\_h_{1}$: First-stage rotor blade height
$a\_h_{2}$: Second-stage rotor blade height
$a\_b$: Rotor blade thickness
$a\_b_{r}$: Rotor blade root thickness
$a\_b_{t}$: Rotor blade tip thickness
$a\_s_{r}$: Rotor blade spacing root
$a\_s_{t}$: Rotor blade spacing tip
$a\_n$: Number of rotor blade teeth
B1: Stator inlet
B2: Stator front blades
B3: Stator rear blades
B4: Stator body
B6: Stator outlet
B7: Stator top surface
B8: Stator bottom surface
$b\_r_{r}$: Stator blade root radius
$b\_r_{t}$: Stator blade tip radius
$b\_\alpha$: Stator blade angle
$b\_\alpha_{r}$: Stator blade root angle
$b\_\alpha_{t}$: Stator blade tip angle
$b\_h$: Stator blade height
$b\_h_{1}$: First-stage stator blade height
$b\_h_{2}$: Second-stage stator blade height
$b\_b$: Stator blade thickness
$b\_b_{r}$: Stator blade root thickness
$b\_b_{t}$: Stator blade tip thickness
$b\_s_{r}$: Stator blade spacing root
$b\_s_{t}$: Stator blade spacing tip
$b\_n$: Number of stator blade teeth
$\delta$: Gap between rotor and stator
$M$: Gas molecule mass
$R$: Universal gas constant
$$T$$: Temperature
$V_{{{\text{mp}}}}$: Most probable velocity
