# Supplementary material for: Structural optimization and flow field analysis of turbomolecular pump based on a new performance prediction algorithm
Source: Sci Rep. 2024 Jun 3;14:12735. doi: 10.1038/s41598-024-63690-9 (PMC11637116; doi:10.1038/s41598-024-63690-9)
Supplement: Supplementary file 1 — Supplementary Information. [file 41598_2024_63690_MOESM1_ESM.docx]

# Supplementary Material

The blade equation of each stage turbine blade row is very vital when establishing simulation calculating model. It can affect accuracy of simulation calculating results. The front blades equation of rotor row is solved under the static coordinate system, which is as shown in Eq. (4). The detailed solving process is as follows.

In Fig. S1, front blades equation of rotor row is got under the moving coordinate system, which is as shown in Eq. (3).


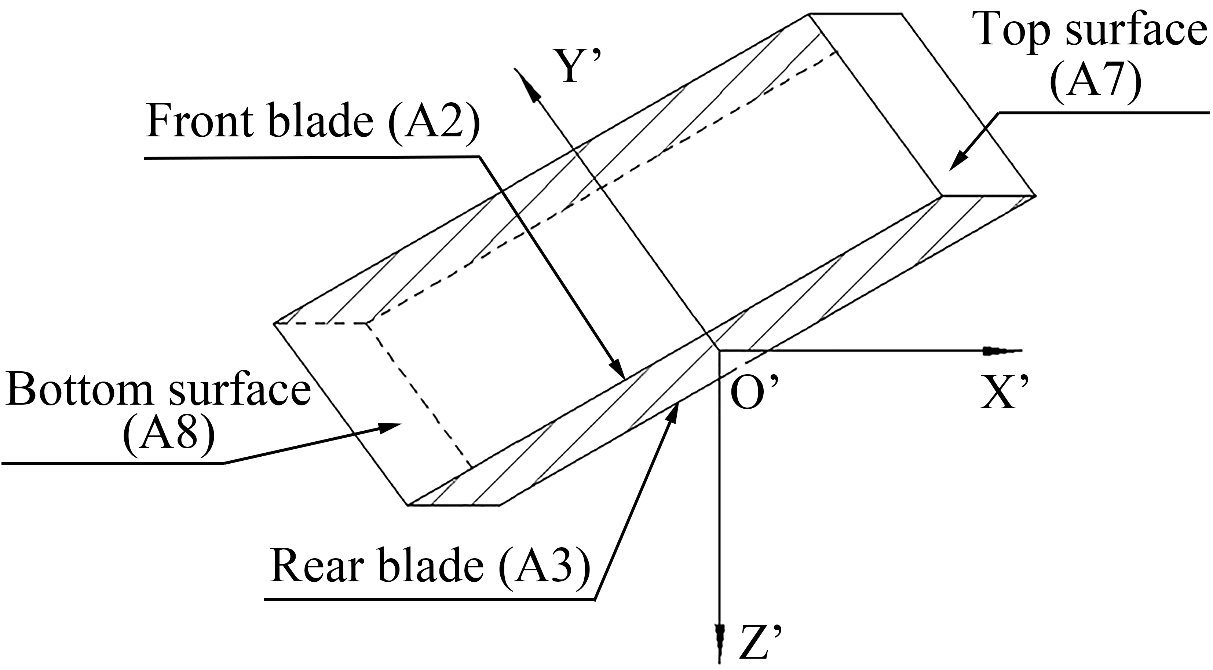


**Figure S1.** A rotor blade in the moving coordinate system.

Combined with Eq. (1) ($z'=z-a\_h/2$ and $x'=x\text{cos}\beta-y\text{sin}\beta$), Eq. (3) can be obtained as follows Eq. (S1):

$x\text{cos}\beta-y\text{sin}\beta+\left(z-\frac{a\_h}{2}\right)\text{cot}a\_\alpha+\frac{a\_b}{2\text{sin}a\_\alpha}=0$ (S1)

Equation (S1) is the blade No. 0 equation when the rotor blade row runs $t$ time by an angle velocity$\omega$. Supposing that blade No. $a\_m$ stands for any blade number, the A2 blade No. $a\_m$ equation is expressed by Eq. (4) when rotor blade row runs $t$ time at an angle velocity$\omega$, as shown in Eq. (S2).

$x\text{cos}\left(\frac{2\pi a\_m}{a\_n}+\omega t\right)-y\text{sin}\left(\frac{2\pi a\_m}{a\_n}+\omega t\right)+\left(z-\frac{a\_h}{2}\right)\text{cot}a\_\alpha+\frac{a\_b}{2\sin a\_\alpha}=0$ (S2)

Solving process of Eq. (7), Eq. (8) and Eq. (9) is almost the same as Eq. (4). Therefore, their solving process is not provided in Supplementary Material.
